# Neophytes may promote hybridization and adaptations to a changing planet

**DOI:** 10.1002/ece3.10405

**Published:** 2023-08-16

**Authors:** Ingmar R. Staude, Jana Ebersbach

**Affiliations:** ^1^ Institute of Biology Leipzig University Leipzig Germany; ^2^ German Centre for Integrative Biodiversity Research (iDiv) Halle‐Jena‐Leipzig Leipzig Germany

**Keywords:** anthropogenic hybridization, biodiversity redistribution, biotic and genetic homogenization, diversification, human footprint, non‐natives

## Abstract

Human activities erode geographic barriers, facilitating hybridization among previously isolated taxa. However, limited empirical research exists on the consequences of introduced species (neophytes) for hybridization and subsequent evolutionary outcomes. To address this knowledge gap, we employed a macroecological approach. First, we examined the spatial and phylogenetic overlap between neophytes and hybrids by integrating the Plants of the World Online database with the Global Naturalized Alien Flora database. Second, leveraging the largest dated plant phylogeny available, we compared diversification rates between genera containing hybrids and neophytes versus those without. Third, focusing on the extensively studied hybrid flora of Britain, we studied the spatial distributions of hybrids in relation to neophyte and native parents, assessing potential adaptations to anthropogenic disturbances and impacts on native species. Overall, our findings highlight positive ties between contemporary biodiversity redistribution and hybridization. Spatially (across countries) and phylogenetically (across genera), neophyte incidence was positively associated with hybrid incidence. Genera comprising both hybrids and neophytes displayed significantly higher diversification rates. Neophyte hybrids primarily occupied areas with a higher human footprint, with limited evidence of hybrids threatening native species throughout their range in more natural habitats. These results challenge the notion that species naturalizations and hybridizations exclusively yield negative outcomes for biodiversity. While it is conceivable that anthropogenic hybridization may facilitate recombination of genetic variation and contribute to conserving genetic diversity in disturbed environments, further research is needed to fully understand these processes.

## Graphical abstract

The human‐mediated breakdown of geographic barriers to gene flow provides previously isolated, genetically distinct taxa with unprecedented new opportunities for hybridization, potentially having a profound impact on biodiversity. We studied the impact of neophyte naturalization on hybrid formation from a macroecological perspective and aimed to gain more insights into its potential evolutionary consequences. Our findings are consistent with the hypothesis that a global reshuffling of biota augments hybridization rates possibly representing not only a threat to diversity, but a process that can promote diversification and adaptations to anthropogenic global change.

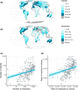

## 1 INTRODUCTION

Hybridization permeates the evolutionary history of plants and is known to be involved in the origin of a significant proportion of plant species (Rieseberg, 1997; Soltis & Soltis, 2009; Stebbins, 1959). Interspecific hybridization describes the process by which crosses between genetically distinct taxa result in the production of viable offsprings that are genetically and often phenotypically distinct (Mallet, 2005, 2007). By promoting novel combinations of parental alleles, hybridization may spur accelerated rates of speciation and so generate biodiversity (Abbott et al., 2013; Anderson & Stebbins Jr, 1954; Marques et al., 2019; Thomas, 2015). Per contra, hybridization can erode the distinctiveness of native and especially endemic species through admixture and introgression with widespread species, leading to genetic homogenization, thereby threatening biodiversity (Daehler & Carino, 2001; Levin et al., 1996; Rhymer & Simberloff, 1996; Todesco et al., 2016). Empirical evidence suggests that hybridization rates may be increasing in the Anthropocene due to the human‐mediated breakdown of geographic and ecological barriers with the naturalization of many plant species outside their historical range (Vallejo‐Marin & Hiscock, 2016). This may have important, but still poorly understood, implications for biodiversity.

More than 13,000 plant taxa have been naturalized (i.e., permanently established) by human activities somewhere on the globe since the year 1492 (referred to as neophytes; Van Kleunen et al., 2015). In an era of globalization with international trade and travel, as well as global warming, the number of neophyte introductions continues to increase with no sign of saturation (Seebens et al., 2017). This global reshuffling of biota is likely to impact the rate of hybrid formation by providing previously isolated, genetically distinct taxa with unprecedented new opportunities for hybridization (i.e., anthropogenic hybridization; Allendorf et al., 2001; McFarlane & Pemberton, 2019; Thomas, 2015; Vallejo‐Marin & Hiscock, 2016). One of the best‐studied hybrid floras in the world, the Hybrid Flora of the British Isles, shows neophytes can contribute markedly to hybrid formation (Stace et al., 2015). Out of the 744 spontaneous hybrids that have arisen on the British Isles, 139 have at least one neophyte parent (c. 19%) and taking hybrid complexes and introduced hybrids into account 33% of the recorded hybrids (301 out of 909) involve introduced taxa (Preston & Pearman, 2015; Stace et al., 2015). These numbers suggest that increasing species naturalizations may be positively associated with hybrid formation and incidence but the extent of such a correlation on a global scale is unknown.

Hybrid formation itself does not necessarily predict evolutionary success of the hybrid taxa. In fact, many of the hybrids of the British Isles have low fertility and occur exclusively with their parental species in transient populations (Preston & Pearman, 2015). At the same time, multiple of these hybridizations involving neophytes have given rise to new species in the last 200 years (Vallejo‐Marin & Hiscock, 2016). While the genetics literature is beginning to see both natural and anthropogenic hybridization as important processes for generating biodiversity (Marques et al., 2019; Thomas, 2015), the more ecologically oriented biodiversity literature tends to see them as processes that threaten native species and generate invasive ones that reduce species diversity (Hamilton & Miller, 2016; Thomas, 2015); both of which can be true (Ellstrand, 2009; Levin et al., 1996; McKinney & Lockwood, 1999). The potential evolutionary consequences of anthropogenic hybridization and whether a propensity for hybridization (with or without neophytes) drives diversification rates over larger time scales remain poorly understood.

Over shorter time scales, hybridization with neophytes is typically assumed to pose a conservation concern (Vilà et al., 2000). Neophyte naturalization and associated hybridizations can reduce the progeny of native species, which may waste important pollen material on unfit hybrids (demographic swamping), and if the fitness of the hybrid exceeds that of the native parent (hybrid vigor), hybrids may displace native and unique genetic variation (genetic swamping; Daehler & Carino, 2001; Levin et al., 1996; Todesco et al., 2016). Conversely, neophytes often exhibit fast rates of growth and reproduction (Mathakutha et al., 2019; Rejmanek & Richardson, 1996), allowing them to thrive in habitats experiencing frequent natural or anthropogenic disturbances (Chytrý et al., 2008; Jauni et al., 2015). Hybrids that have inherited rapid growth from the neophyte and some local adaptations from the native parent, might further be adapted to habitat disturbances where natives might otherwise be lost. Moreover, if hybrid occurrences are limited to preferentially disturbed areas, that is, places with a high human footprint, they may pose little additional risk to native species outside these areas. Anthropogenic hybridization could therefore also act to fill novel ecological opportunities while maintaining evolutionary potential (Hamilton & Miller, 2016).

Here we (1) investigate whether neophytes may drive hybrid formation globally and (2) gain insights into the potential long‐ and short‐term evolutionary consequences of anthropogenic hybridization. For (1), we integrated the Plants of the World Online (Govaerts et al., 2021; POWO) and Global Naturalized Alien Flora databases (van Kleunen et al., 2019; GloNaF) to investigate the spatial and phylogenetic overlap in the incidence of neophytes and hybrids, asking whether countries and genera with more neophytes also have more hybrids. For (2), we use the largest dated phylogeny for vascular plants to date (Jin & Qian, 2019) to compare diversification rates between genera with hybrids and neophytes to those with neither, asking whether a propensity to hybridize and naturalize is associated positively to diversification. Further, we ask whether neophyte hybrids may threaten or help preserve native genetic variation (that might otherwise be lost from the extirpation of native parental species) in the short term. For this, we used the hybrid flora of Britain (Stace et al., 2015) together with occurrence records from the Botanical Society of Britain & Ireland (BSBI) to compare the geographic distribution of natives, neophytes, and hybrids in relation to the human footprint. Together, our study provides a macroecological perspective of how biodiversity redistribution may impact hybridization and evolutionary processes.

## 2 METHODS

R code for all data carpentry, analyses and figures, and all data are available on GitHub at: https://github.com/istaude/neophytes‐hybrids. We employed a Bayesian modeling framework implemented via the brms package (Bürkner, 2017) for all statistical analyses. For all analyses, we use the 95% credible interval to determine statistical clarity (Dushoff et al., 2019).

### 2.1 Spatial overlap between hybrids and neophytes

We tested the relationship between the number of hybrids and the number of naturalized neophytes across Taxonomic Database Working Group (TDWG) Level 3 regions. For this, we used the World Checklist for Vascular Plants (WCVP February 2021; WCVP, 2021) to retrieve accepted interspecific hybrids (excluding artificial hybrids), which we identified via WCVP's “species_hybrid” column. We used the Plants of the World Online (POWO; POWO, 2021) database to retrieve the global geographic distribution of hybrids at the level of botanical countries (TDWG Level 3). We used the Global Naturalized Flora (GloNaF; van Kleunen et al., 2019) to retrieve the geographic distribution data of the world's naturalized neophyte flora; also at the level of botanical countries. Seven TDWG Level 3 regions were incorrectly coded in GloNaF as numeric (e.g., a region in Indonesia has the value 2 and in New Guinea the value 29), we excluded these as we were not able to find the correct TDWG Level 3 code in these cases. To prevent regressing hybrids on hybrids, we excluded hybrids that are neophytes, which we identified via GloNaF's “hybrid” column. Further, we only included accepted neophyte species (identified via GloNaF's “name_status” column) and those that naturalized (i.e., we excluded those listed as aliens—species for which it is unclear whether they are established, self‐sustaining, and reproducing, which we assume is a prerequisite for hybridization; identified via GloNaF's “status” column). Botanical countries with no data on either hybrids or neophytes were excluded in this analysis, as this represents more likely a gap in data mobilization than true absence.

Any relationship between hybrid and neophyte incidence may be affected by taxonomic effort. Furthermore, this bias is likely stronger for hybrids. Neophytes are often widespread species and are therefore easier to identify and record than hybrids where characteristics differentiating the hybrid from the parentage need to be known or easily recognizable (which is often easier for young generation hybrids). We attempted to account for taxonomic effort in two ways: (1) as per capita GDP, and (2) the number of taxonomists active in a country. We obtained per capita GDP via the R package WDI (Arel‐Bundock, 2021) for all countries (querying “NY.GDP.PCAP.KD”) for the period 1960–2020. We averaged GDP across this period. Since GDP data are at the scale of political countries, we cross‐referenced GDP data to botanical countries: if multiple botanical countries occurred within one political country, they obtained the same GDP value; if multiple political countries had territory in one botanical country, the botanical country obtained the average GDP value. To quantify the number of taxonomists, we followed the approach of Rodrigues et al. (2010). We counted the number of authors first describing and publishing plant species in a given botanical country, assuming that an author worked in a given area if at least one of the species she or he studied was present. For species with very large ranges, this produces a spillage effect. We therefore calculated for each taxon the number of botanical countries in which it occurs and weighted the authors by the reciprocal of the average range size of the species they described in a given botanical country. Moreover, larger countries may not only have more neophytes but also more hybrids, therefore, we also controlled for country area. Finally, we calculated the ratio of neophytes and hybrids to native species, and tested whether accounting for species richness in botanical countries affects the relationship between hybrid and neophyte incidence, again accounting for the above potential confounding variables. We use two Gaussian models,

$$h_{i}∼\text{Normal}\;\left(\mu_{i},\sigma \right)$$

$$\mu_{i}=\alpha +\beta_{n_{i}}n_{i}+\beta_{e_{i}}e_{i}+\beta_{t_{i}}t_{i}+\beta_{a_{i}}a_{i}$$

where $h_{i}$ is the number of hybrids (or ratio of hybrids to natives) in a given botanical country, $n_{i}$, $e_{i}$, $t_{i}$ and $a_{i}$ are the number of neophytes (or ratio of neophytes to natives), economic status, taxonomic effort in and area of that botanical country, respectively.

### 2.2 Phylogenetic overlap between neophytes and hybrids

We tested the relationship between the incidence of neophytes and hybrids across plant genera. Because genera with many species are likely to comprise more neophytes and hybrids, we controlled for species richness within genera. We used the WCVP to count the number of accepted species per genus, excluding varieties, subvarieties, convarieties, forms, subforms, subspecies, and hybrids. We also used WCVP data to count the number of accepted hybrids per genus. GloNaF data were used to count the number of neophyte species per genus, again excluding neophyte hybrids to avoid regressing hybrids on hybrids. The number of accepted species per genus was used to standardize the number of hybrids and neophytes per genus. In some cases, these ratios exceeded 1. For hybrids, for example, 18 accepted species and 26 hybrids were counted for *Equisetum* L. (Equisetaceae). For neophytes, this ratio exceeded 1 for one genus (*Robinia* L. [Fabaceae], four accepted species, six neophytes out of which two were varieties; we included neophyte varieties, as their naturalizations may equally present opportunities for hybridization). We joined WCVP hybrid genera and GloNaF neophyte genera, using the rWCVP and rWCVPdata package (Brown et al., 2023) to harmonize taxonomies. To assess the phylogenetic overlap between the number and ratio of neophytes and hybrids we use two Gaussian models,

$$h_{i}∼\text{Normal}\;\left(\mu_{i},\sigma \right)$$

$$\mu_{i}=\alpha +\beta_{n_{i}}n_{i}$$

where $h_{i}$ is the number of hybrids (or ratio of hybrids to natives) in a given genus and $n_{i}$ is the number of neophytes (or ratio of neophytes to natives) in that genus. We also calculated the phylogenetic signal in the number of hybrids and neophytes to understand whether any relationship between the two is limited to certain clades or extends throughout the plant tree of life. We calculated the phylogenetic signal as Blomberg's *K* (which ranges from 0 to infinity, where 0 indicates no phylogenetic signal) using the phytools package (Revell, 2012) and a genus‐level vascular plant phylogeny of Molina‐Venegas et al. (2021) (which is based on an extension of the seed plant phylogeny of Jin & Qian 2019 and Smith & Brown 2018).

### 2.3 Diversification rates in genera with and without hybrids and/or neophytes

To compare diversification rates among genera with and without neophytes and hybrids, we first split genera into four groups: Genera including (1) neither hybrids nor neophytes, (2) no hybrids but neophytes, (3) hybrids but no neophytes, and (4) both hybrids and neophytes. We then obtained data on genus age from V.PhyloMaker (data: nodes.info.1, column: rn.bl; Jin & Qian, 2019). Net diversification rates per genus were estimated using the method‐of‐moments estimator for stem‐group ages, which requires only stem‐group ages, species richness of genera, and an assumed ratio of extinction (Magallon & Sanderson, 2001; Scholl & Wiens, 2016). We calculated diversification rates within genera as log(SR_G_*(1−*e*) + *e*)/age, where SR_G_ is the number of species in a genus and *e* is the relative extinction fraction. We assumed a constant relative extinction fraction of 0.9, as in Magallon & Sanderson, 2001: At relative extinction rates greater than 0.9, it is suggested that speciation and extinction events would have to occur at a rate of more than one per million years, which is considered unlikely given published estimates of the frequency of these events (Harris & Davies, 2016). The method‐of‐moment estimator assumes a constant extinction rate, overlooking potential variations across different time periods, clades, and geographic regions. Yet, it is widely used and produces comparable outcomes to other diversification rate estimation methods (Mitchell & Whitney, 2021), making it suitable for our broad‐scale phylogenetic analysis due to its relatively simple data requirements (Scholl & Wiens, 2016). We tested if results are robust to assuming a different value for e (i.e., *e* = 0.5; Figure S1). Monotypic genera (e.g., *Garberia* A.Gray, Asteraceae) have a diversification rate of zero, these occurred exclusively in Groups 1 and 2, and we tested whether our analysis is robust to excluding these. Furthermore, 17 genera had extremely high diversification rates (e.g., the relatively young but very diverse genus *Phreatia* Lindl. [Orchidaceae] with 840 spp. per Myr; rn.bl = 0.00371 and n. spp. = 217); we again tested if results are robust to excluding these extreme rates (Figure S1). Our model that tests for differences in diversification rates between genera is,

$$d_{i}∼\text{Normal}\;\left(\mu_{i},\sigma \right)$$

$$\mu_{i}=\alpha +\beta_{\text{group}\left[i\right]}$$

where $d_{i}$ is the log10‐transformed net diversification rate per genus and $\beta_{\text{group}\left[i\right]}$ indicates the respective group (1–4). We calculated all pairwise contrasts between the four groups by calculating the posterior difference in mean diversification rates between groups, implemented via the emmeans package (Lenth, 2022). Since the posterior difference between groups is in the log10‐scale, this gives a ratio of diversification rates between groups after back‐transformed to the original scale.

We note several limitations of this analysis: (1) diversification rates include a longer time span and past than it is likely that neophytes (ad 1497) are a proximate cause; (2) the extent to which hybridization has played a role in past speciation events is currently unclear for many genera as it requires in depth molecular investigation. Keeping these aspects in mind, we propose that (1) specific traits and characteristics can make species more prone to become neophytes, where these traits may have also historically allowed genera to diversify faster, making them useful in the recent past and perhaps also in the future; (2) current propensity of individual plant genera to form hybrids can provide valuable clues regarding the significance of hybridization in the evolutionary history of a lineage (e.g., in *Saxifraga* L. [Saxifragaceae], a genus known for its hybridization propensity, six species have been documented to have arisen from hybridization Ebersbach et al. (2020)).

### 2.4 Comparing the distribution of hybrids with their neophyte and native parent in Britain

To test for differences in the geographic distribution of hybrids, and their native and neophyte parent, in relation to habitat disturbance, we used data from the Hybrid flora of the British Isles and the Botanical Society of Britain & Ireland (BSBI). We obtained the list of spontaneous British hybrids in Preston & Pearman, 2015 that involved a neophyte and native parent personally from Christopher Preston. This list included 86 hybrids and the names of the parent species. For each triplet (hybrid, native, and neophyte) on this list, we queried geographic distribution data from BSBI (https://database.bsbi.org/maps/), more specifically the Taxon‐hectad validation (NBN record cleaner ruleset). This dataset provides the hectad letter codes of the British Ordnance Survey National Grid, for the hectads (i.e., grid cells with a size of 10 x 10 km square) in which taxa occur on the British Isles.

We obtained the human footprint index (HFI) raster data from the Last of the Wild v3 dataset at a spatial resolution of ~1 km (https://sedac.ciesin.columbia.edu/data/set/wildareas‐v3‐2009‐human‐footprint; Venter et al., 2016). We re‐projected the hectad polygons from the British Ordnance Survey National Grid (https://github.com/OrdnanceSurvey/OS‐British‐National‐Grids) to the coordinate reference system of the HFI raster layer (Mollweide projection), to retrieve for each hectad, HFI values, using the sf package (Pebesma, 2018). We then used the hectad polygons to extract HFI values per hectad using the terra package (Hijmans, 2022), and calculated the mean across all the ~1 km^2^ HFI grid cells within a hectad to obtain one mean HFI value per hectad. We joined these mean values to the BSBI species distribution data using the hectad letter code. We included only hybrids that occupied more than 10 hectads. Only 29 of the 86 hybrids with one native and neophyte parent on Preston's list met this criteria. Median number of occupied hectads was 34 for hybrids, 2829 for natives, and 1096 for neophytes.

To test statistically for differences in HFI, $f_{i}$, between hybrid, native and neophyte parent ($\beta_{\text{type}\left[i\right]}$), we predicted HFI with these categories, including a varying intercept ($\alpha_{\text{triplet}\left[i\right]}$) and slope ($\beta_{\text{type}\left[i\right],\text{triplet}\left[i\right]}$) for triplet (i.e., the complex to which hybrid, native and neophyte belong). Our model is,

$$f_{i}∼\text{Normal}\;\left(\mu_{i},\sigma \right)$$

$$\mu_{i}=\alpha_{\text{triplet}\left[i\right]}+\beta_{\text{type}\left[i\right],\text{triplet}\left[i\right]}$$

We calculated all pairwise contrasts between the three types (hybrid, native, and neophyte) by calculating the posterior difference in mean HFI between groups, implemented via the emmeans package (Lenth, 2022).

## 3 RESULTS

### 3.1 Spatial overlap between neophyte and hybrid occurrence

**FIGURE 1:**
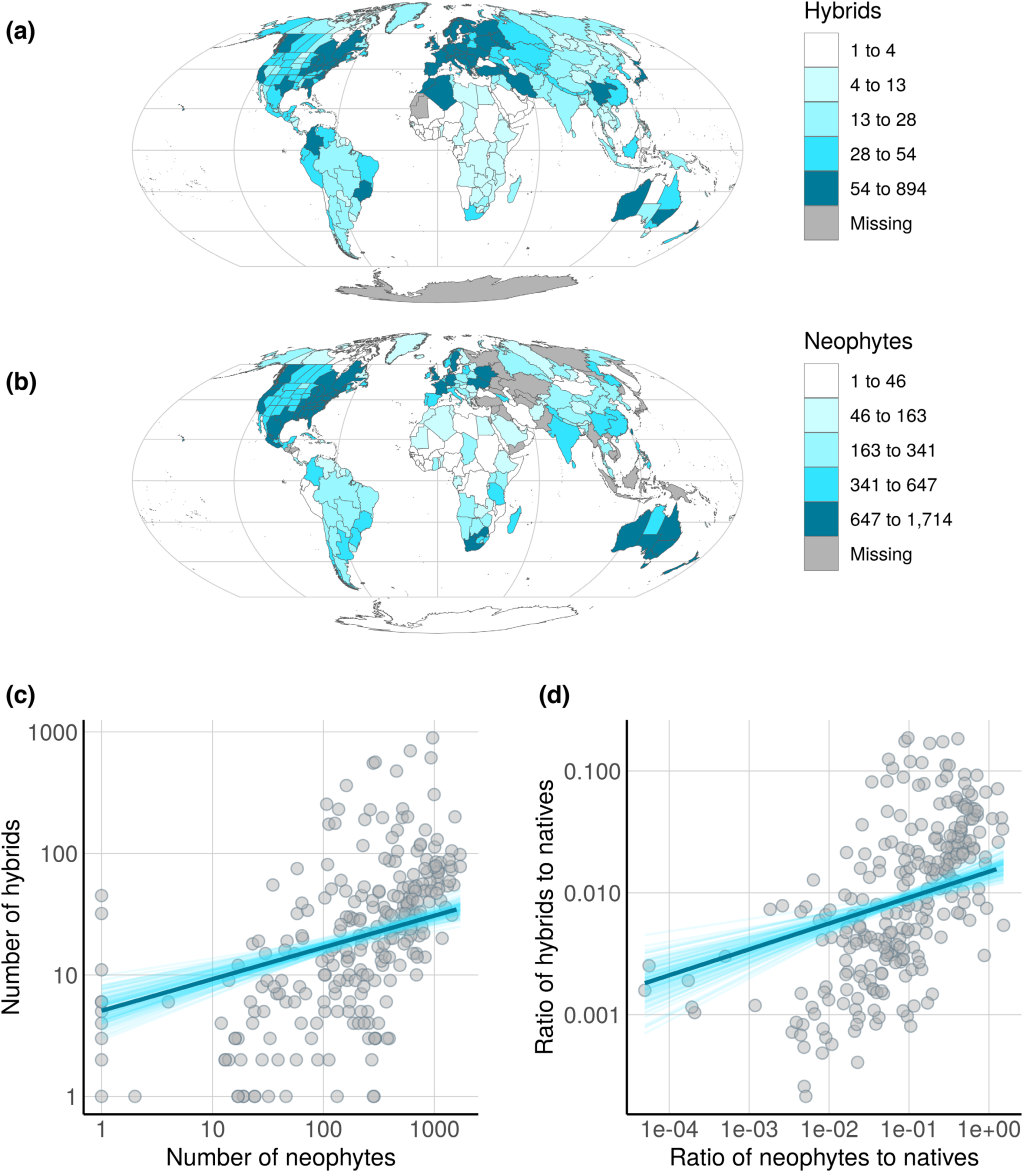
Spatial overlap between neophyte and hybrid incidence. Geographic distribution of the world's (a), hybrids and (b), neophyte naturalizations. Map is in Mollweide projection and colored by quantiles. (c) Botanical countries with more neophytes have more hybrids, holding GDP per capita, taxonomic effort and country size at their mean. (d) Similarly the ratio of hybrids to natives is higher in botanical countries with higher ratios of neophytes to natives. Each shaded circles represent a botanical country. Dark blue line represents the marginal slope, conditional on the other variables held at their respective mean. Light blue lines represent 100 draws from the posterior distribution of fit lines. Both predictor and response variable were log‐transformed and axes are on the log10 scale.

A total of 6818 accepted hybrids are listed in WCVP. Comparing the geographic distribution of hybrids with the naturalized ranges of 11,987 accepted neophyte species listed in GloNaF, revealed substantial spatial overlap (Figure 1a,b). Botanical countries with more neophyte naturalizations also had more hybrids. The positive association between the number of hybrids and neophytes persisted after accounting for GDP per capita, a proxy for taxonomic effort, and country size (slope (*b*) = 0.26, standard deviation (SD) = 0.05; predictors and response were log10‐transformed, Figure 1c). For every 20% increase in the number of neophytes, the number of hybrids increased by about 5%, holding the other variables constant. A similarly positive and statistically clear association was found between the ratio of the number of neophytes to natives and the ratio of hybrids to natives, holding the other variables constant (*b* = 0.21, SD = 0.04; predictors and response were log10‐transformed; Figure 1d).

Given that the parentage of hybrids is typically unrecorded and existing data are often not mobilized, we were unable to calculate the true percentage of hybrids involving neophytes. To further substantiate the above spatial association between neophytes and hybrids, we estimated the probability that a species naturalized somewhere would encounter a species of the same genus (often a necessary condition for hybridization). We estimate that of the 119,917 naturalization events recorded in GloNaF (at the TDWG Level 3; botanical countries), congeners are present in the invaded botanical country in about 50% (*n* = 60,109). We further estimate that about 73% (*n* = 8705) of the neophytes in GloNaF could theoretically have encountered a species of the same genus in at least one of the regions where they naturalized.

### 3.2 Phylogenetic overlap between genera that include neophytes and hybrids

**FIGURE 2:**
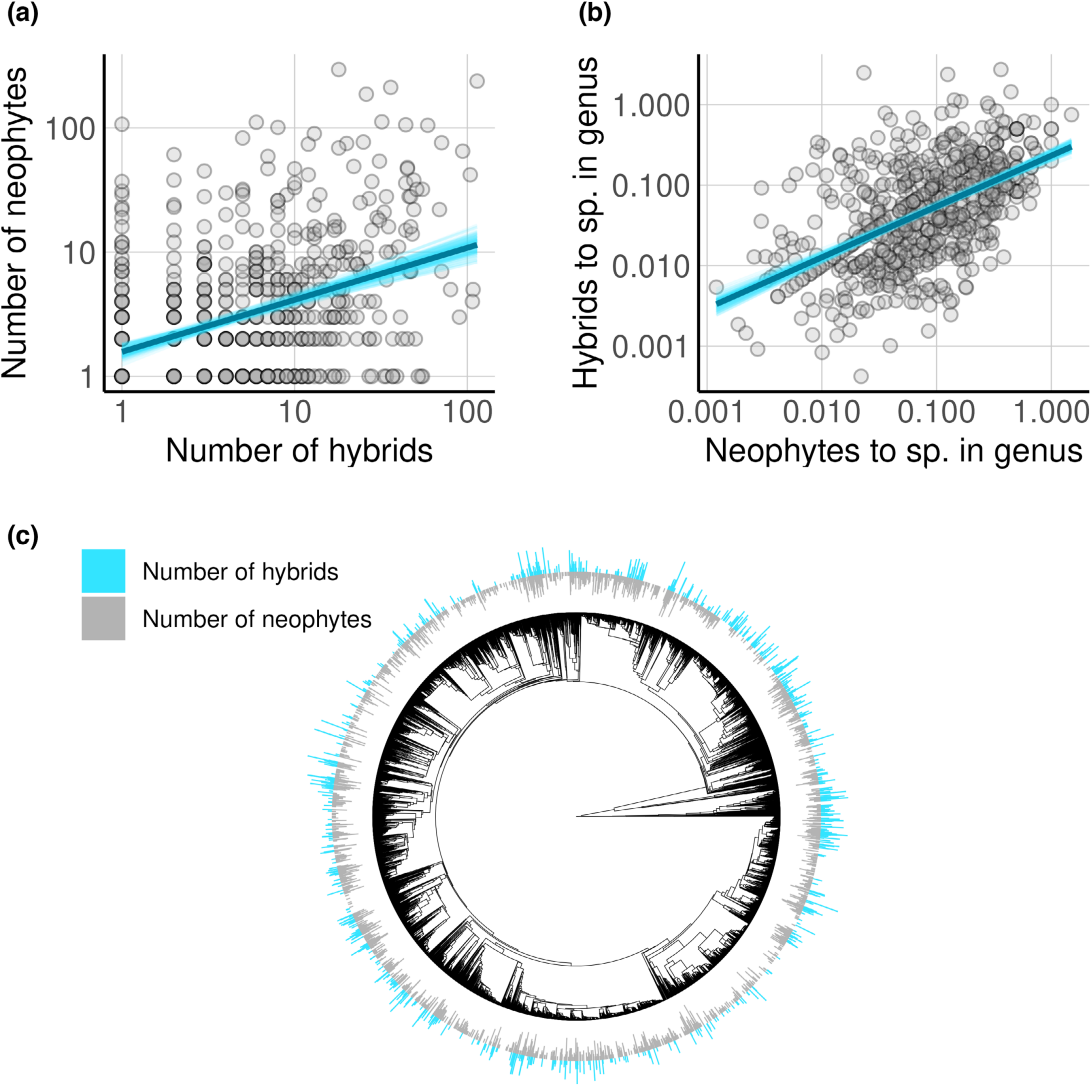
Phylogenetic overlap between neophytes and hybrids. Genera with both neophytes and hybrids show a positive association between neophyte and hybrid (a), numbers and (b), ratios relative to the number of accepted species per genus. Note that there are many genera containing neophytes that do not have hybrids, and vice versa. (c) Incidence of genera with both hybrids and neophytes was scattered across the phylogenetic plant tree of life. Tree includes 9203 genera: genera without neophytes and hybrids, and genera with neophytes and hybrids. Gray and blue rings represent number of neophytes and hybrids at each tip of the circular genus‐level vascular plant phylogeny. For better visibility, the columns in the ring have a larger width than the branches of the tree so that these do not match entirely.

The world's neophytes listed in GloNaF are distributed among 2872 genera; the world's hybrids listed in POWO are distributed among only 887 genera. Many genera with neophytes therefore do not contain hybrids indicating that many neophyte naturalizations may not increase the rate of hybrid formation. Still, there is overlap. Of the 877 genera with hybrids, 75% (*n* = 663) also included neophytes. These 663 genera accounted for more than half of all neophyte species (56%; *n* = 6663), and naturalization events (57%; *n* = 68,508) and were scattered across the vascular plant tree of life (Figure 2c). For the genera containing both hybrids and neophytes, we asked whether there is a relationship between the amount of neophytes and hybrids. There was a statistically clear positive association between the number of hybrids per genus and the number of neophytes (*b* = 0.43, SD = 0.04; predictor and response were log10‐transformed, Figure 2a). However, this might be confounded by the total number of species per genus. We therefore calculated the ratio of hybrids, and separately of neophytes, to the number of accepted species per genus. The two ratios were also clearly positively associated (*b* = 0.64, SD = 0.04; predictor and response were log10‐transformed, Figure 2b). For every 20% increase in the ratio of neophytes, the ratio of hybrids increased by about 12%. Examples of genera with both a high ratio of neophytes and hybrids include: *Equisetum* (26 hybrids and nine neophytes relative to 18 species), *Populus* L. (Salicaceae, 29 hybrids and 21 neophytes relative to 56 species), and *Rumex* L. (Polygoncaceae, 105 hybrids and 63 neophytes relative to 195 species). Examples where the prevalence of neophytes and hybrids varies greatly were found in Orchidaceae, such as *Dactylorhiza* Neck. Ex Nevski, which according to WCVP includes 43 accepted species, 107 hybrids, but only one neophyte. Both the frequency of hybrids and neophytes per genus did not exhibit a strong phylogenetic signal (Figure 2c; Blomberg's *K* = 0.088 and 0.03, respectively), suggesting that the relationship between hybrids and neophytes is not restricted to specific clades but largely applies across the plant tree of life.

### 3.3 Diversification rates in genera with and without hybrids and neophytes

**FIGURE 3:**
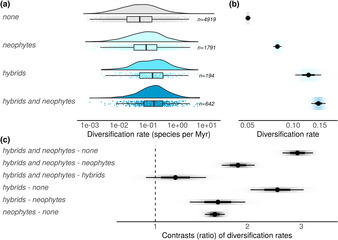
Genera with hybrids and neophytes diversify faster than those without. (a) Raw data (density plots, box plot, and jittered data points) of diversification rates within genera including neither hybrid nor neophyte, only neophytes, only hybrids, and both neophytes and hybrids. (b) Posterior distribution of the mean diversification rate per group. (c) Ratios of the mean diversification rate between groups. Point and lines in c and b are the median and its 66% and 95% credible interval of the posterior distribution. *X*‐axes are on the log10 scale.

Diversification rates were clearly higher in genera with neophytes or hybrids compared to those with neither. Genera that included both hybrids and neophytes had an average diversification rate of 0.15 species per million years, which was about three times higher than those without that had a diversification rate of 0.05 (ratio = 2.91, SD = 0.17, Figure 3). However, this difference was not driven by neophytes. Genera with only hybrids had clearly higher diversification rates than those with only neophytes (ratio = 1.6, SD = 0.17), and although genera with both neophytes and hybrids tended to have the highest diversification rates, these did not differ substantially from those of genera including only hybrids (ratio = 1.16, SD = 0.13). Hybridization and the propensity to become a neophyte may therefore independently be associated with higher diversification rates.

### 3.4 Comparing the distribution of hybrids with their neophyte and native parent in Britain

**FIGURE 4:**
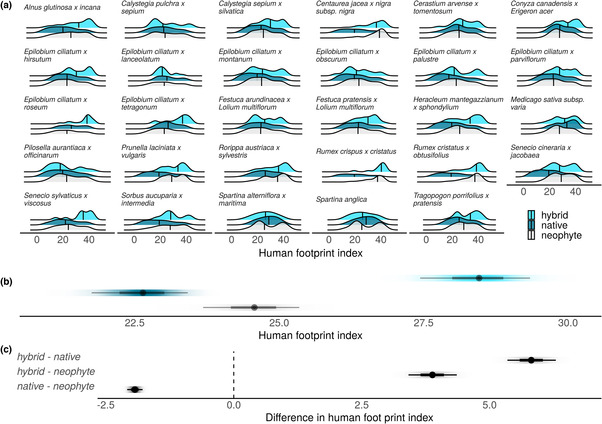
British hybrids are distributed over areas with a higher human footprint than their native and neophyte parent, on average. (a) Raw data of human footprint index (HFI) values across the geographic distribution of hybrid, native, and neophyte in Britain (i.e., England, Wales, and Scotland). Geographic distribution was recorded at the hectad scale on the British Ordnance Survey National Grid. HFI ranges from 0 to 50. (b) Estimated mean HFI for hybrids (light blue), natives (dark blue), and neophytes (gray). (c) Difference in the estimated mean HFI between hybrid, native, and neophyte. Dots, thick and thin lines represent the median, 66% and 95% credible interval of the posterior distribution. Color and gray shading in (b and c) respectively, represents the density of posterior distribution.

Focusing on the relatively well‐documented British hybrid flora, we found that hybrids occurred on average in areas with a higher human footprint index (HFI) than their native and neophyte parent. Hybrids occurred on average in areas with a HFI of 28.5, while natives and neophyte distributions averaged 22.6 and 24.6, respectively. These differences were statistically clear for all pairwise comparisons (hybrid vs. native: *d* = 5.81, 95% CI [5.33, 6.27]; hybrid vs. neophyte: *d* = 3.88, 95% CI [3.40, 4.34]; Figure 4). Neophytes were also associated with more human‐dominated habitats than natives (native vs. neophyte: *d* = −1.93, 95% CI [−2.09, −1.79]). We also found evidence that hybrids mostly did not occur in the more natural habitats that their native parents prefer. Twenty‐four out of 26 hybrids had distributions that did not include the minimum HFI of their native parents. On the other hand, only five out of 26 hybrids had a higher maximum HFI than either their neophyte or native parent. The comparison of geographic distributions was unbalanced with hybrids occupying on average much fewer hectads than their native and neophyte parent (hybrids: 34, neophytes: 1096, natives: 2829).

## 4 DISCUSSION

Here we found that species naturalization is positively associated with hybrid incidence, both spatially and phylogenetically. Accounting for potential confounds, countries and genera with more neophytes had more hybrids (Figures 1 and 2). We also found that genera containing both hybrids and neophytes had higher rates of diversification than genera containing neither (Figure 3). The geographic distribution of neophyte‐native hybrids further revealed that hybrids occurred, on average, in areas with a larger human footprint than their parents (Figure 4). Our findings are consistent with the hypothesis that a global reshuffling of biota affects hybridization rates (Vallejo‐Marin & Hiscock, 2016), possibly representing not only a threat to diversity, but also a process that can promote diversification and adaptations to environmental challenges, particularly anthropogenic stressors.

Our finding that neophyte naturalization is associated positively with hybrid incidence globally echoes previous, more localized studies that find neophyte richness correlates with hybrid richness (Guo, 2014), and that neophyte‐native hybrids can constitute a sizeable part of a country's hybrid flora (Guo, 2014; Preston & Pearman, 2015). The global association between neophytes and hybrids, as of our study, is however only correlative. While we control for indicators of sampling effort, such as GDP per capita and taxonomic effort, country size, and floral richness, other confounding variables may play a role, as even the comprehensive POWO data are likely subject to knowledge biases. Mobilization of data on which taxon served as the maternal and paternal parent could greatly help quantify the true proportion of neophytes involved in hybridization. Nonetheless, the high probability of a neophyte encountering a congener in a given botanical country (we calculated 73%) underscores the potential role neophytes may play in hybrid formation. Plainly, not all species hybridize, and some taxa are much more likely to hybridize than others (Ellstrand et al., 1996; Whitney et al., 2010). But assuming that 9%–25% of plant species hybridize (Mallet, 2005; Whitney et al., 2010), this could mean that of the 73% of neophytes that could encounter a congener somewhere, some 788 to 2188 hybridization events (i.e., 11,987 × 0.73 × 0.25/0.09) may follow from the species introductions to date. Some of these have clearly realized, as evidenced in the hybrid flora of the United States and the British Isles where 185 out of 1126 and 301 out of 909 hybrids, respectively, involve neophytes (Guo, 2014; Preston & Pearman, 2015).

Our examination of the phylogenetic overlap between neophytes and hybrids at the plant genus level provides additional evidence for the potential role of neophytes in hybridization. While only 23% of neophyte genera contained hybrids, a striking 75% of genera with hybrids contained neophytes. On the one hand, this suggests that clearly some of the global reshuffling of species is without consequences for hybridization. On the other hand, genera with hybrids generally also contained neophytes, indicating a possible link between the two. Indeed, in genera with both hybrids and neophytes, we found evidence that more neophytes per species were associated with more hybrids per species. This finding aligns with previous studies that revealed genera involved in native‐neophyte hybridization in Britain comprise more neophytes, accounting for confounding factors such as the effort by which genera are studied (Daehler & Carino, 2001). Given the geographic and phylogenetic overlap observed here, we propose that species naturalizations of some genera can increase, perhaps even considerably, hybrid formation. If hybridization involving neophytes poses a threat to biodiversity, the phylogenetic overlap of hybrids and neophytes in this study may indicate genera at particular risk of introgression, displacement or extinction of parental species and provide a starting point for targeted efforts to limit the spread of such neophytes. Conversely, if these hybridizing neophytes promote biodiversity, the geography of their spread can be used to model and understand contemporary hybrid zones and their roles in diversification.

Hybridization's consequences, especially with neophytes involved, remain a contentious topic (Todesco et al., 2016; Vallejo‐Marin & Hiscock, 2016). Human‐assisted introductions of species are widely believed to homogenize the world's biota, and hybridization is considered to contribute to genetic erosion (Daru et al., 2021; Hamilton & Miller, 2016; Levin et al., 1996; Rhymer & Simberloff, 1996; Yang et al., 2021). Our study revealed that genera containing both neophytes and hybrids show higher past diversification rates than genera with neither, suggesting hybridization and naturalization propensity confer evolutionary benefits. A propensity for hybridization has long been recognized as a potential driving force of diversification, facilitating evolutionary processes such as adaptive introgression and allopolyploidization (Abbott et al., 2013; Mitchell & Whitney, 2021). For instance, repeated backcrossing of an interspecific hybrid with one of its parents can lead to the introgression and recombination of genetic variation (Marques et al., 2019), where combinations of divergent alleles (as is likely in hybridizations involving neophytes) may provide a particularly good substrate for speciation (Comeault & Matute, 2018; Marques et al., 2019). Furthermore, the observed positive association between neophytes presence and high diversification rates could point to an advantageous role of common neophyte traits in diversification, suggesting that hybridization with neophytes could have positive outcomes for biodiversity. For example, in *Mimulus*, *Senecio*, and *Spartina*, hybrid speciation events involving neophytes are well‐documented (Vallejo‐Marin & Hiscock, 2016). But other intrinsic factors promoting diversification might coincide with hybridization incidences (e.g., perennialism, woodiness; Mitchell et al., 2019), complicating mechanistic understanding. Given the ties between biodiversity redistribution and increased hybridization rates, a comprehensive understanding of the evolutionary consequences must be at the core of informed biodiversity management.

To test whether the transfer of adaptive genetic variation plays a role as an evolutionary process, we tested whether neophyte‐native hybrids occur in habitats with higher disturbance intensities than their parents. We found that neophyte‐native hybrids in Britain occurred, on average, in areas with a larger human footprint than their parents, consistent with early studies that propose hybrids thrive in disturbed habitats (Anderson, 1948; Wiegand, 1935). One possible explanation for this pattern is indeed that it reflects a transfer of adaptive genetic variation, in that hybrids inherit certain beneficial growth characteristics of the neophyte and some local adaptations of the native, allowing hybrids to survive and spread in perturbed areas (Guo, 2014). However, because neophytes are generally more common in disturbed habitats and habitat disturbance can bring two parental species into close proximity, favoring cross‐pollination rates, it is possible that neophyte‐native hybrids preferentially occur in disturbed habitats for these reasons alone (Daehler & Carino, 2001; Vilà et al., 2000). Moreover, sampling biases may play a role, as late‐generation hybrids might go more frequently unrecorded, especially in areas with a smaller human footprint. Yet population genetic studies with standardized field sampling, where such sampling biases are unlikely to play a major role, also show that hybrid individuals, introgression, and admixed populations are much more common in anthropogenic landscapes (Hoban et al., 2012; Thompson et al., 2010), consistent with our findings. While we cannot disentangle these possible explanations here, it is evident that hybrids of neophyte × native parentage do occur in disturbed areas. This suggests these neophyte‐native hybrids are to some degree adapted to disturbances characteristic for the Anthropocene. Furthermore, the preferential occurrence of hybrids in disturbed areas suggests they do not yet compete with and threaten native species in more natural habitats (Ruhsam et al., 2023), but instead may help fill environments unsuitable for many native species.

## 5 CONCLUSION

Here we studied the impact of neophyte naturalization on hybrid formation from a macroecological perspective and aimed to gain more insights into its potential consequences. The patterns we found suggest species naturalization concur with increased hybrid incidence and that hybridization propensity may be positively linked to diversification. In light of these findings and given the recent history of human‐mediated species introductions, we predict that hybrid formation is likely to increase over the next centuries, especially in countries, where international trade expands and economies grow. We further suggest novel species naturalizations and associated hybridization events need not have only negative evolutionary outcomes, such as biotic and genetic homogenization. Against a backdrop of rapid global change, these processes may also promote recombination of genetic variation and adaptation to disturbed environments. Yet we emphasize that our results are only correlative, and that confounding variables such as sampling and knowledge biases should always be considered when interpreting such results. It will be essential to study the mechanisms underlying hybridization from species naturalization in future work. Contemporary biodiversity redistributions and associated hybridizations provide a fascinating natural experiment to understand nature's strategy to adapt to the novel environments of a human‐dominated planet.

## AUTHOR CONTRIBUTIONS

**Jana Ebersbach:** Conceptualization (supporting); formal analysis (supporting); investigation (supporting); visualization (supporting); writing – original draft (supporting). **Ingmar R. Staude:** Conceptualization (lead); data curation (lead); formal analysis (lead); investigation (lead); methodology (lead); visualization (lead); writing – original draft (lead).

### OPEN RESEARCH BADGES

This article has earned an Open Data badge for making publicly available the digitally‐shareable data necessary to reproduce the reported results. Data for the project is stored here: https://github.com/istaude/neophytes‐hybrids; Publicly available data are available at POWO: https://storage.googleapis.com/kew‐dev‐backup/world_checklist_names_and_distribution_feb_21.zip; GloNAF: https://idata.idiv.de/DDM/Data/ShowData/257; Human footprint: https://sedac.ciesin.columbia.edu/data/set/wildareas‐v3‐2009‐human‐footprint; British Ordnance Survey national grid is available at: https://github.com/OrdnanceSurvey/OS‐British‐National‐Grids.

## Supporting information


Figure S1.

## Data Availability

Raw and summary data, as well as R code for all data carpentry, analyses and figures are available on GitHub at: https://github.com/istaude/neophytes‐hybrids.
