# Supplementary material for: Neophytes may promote hybridization and adaptations to a changing planet
Source: Ecol Evol. 2023 Aug 16;13(8):e10405. doi: 10.1002/ece3.10405 (PMC10427993; doi:10.1002/ece3.10405)
Supplement: Supplementary file 1 — Figure S1. [file ECE3-13-e10405-s001.docx]

**Supplementary Material**

for

*Neophytes may promote hybridization and adaptations to a changing planet*

Ingmar R. Staude^1,2^ & Jana Ebersbach^1,2^

1. *Institute of Biology, Leipzig University, Leipzig, Germany*
2. *German Centre for Integrative Biodiversity Research (iDiv) Halle-Jena Leipzig, Leipzig, Germany*

**Content**

**Supplementary Figures:**

**Figure S1:** Diversification rates in relation to whether genera include hybrids and/or neophytes are robust to different methodological procedures.


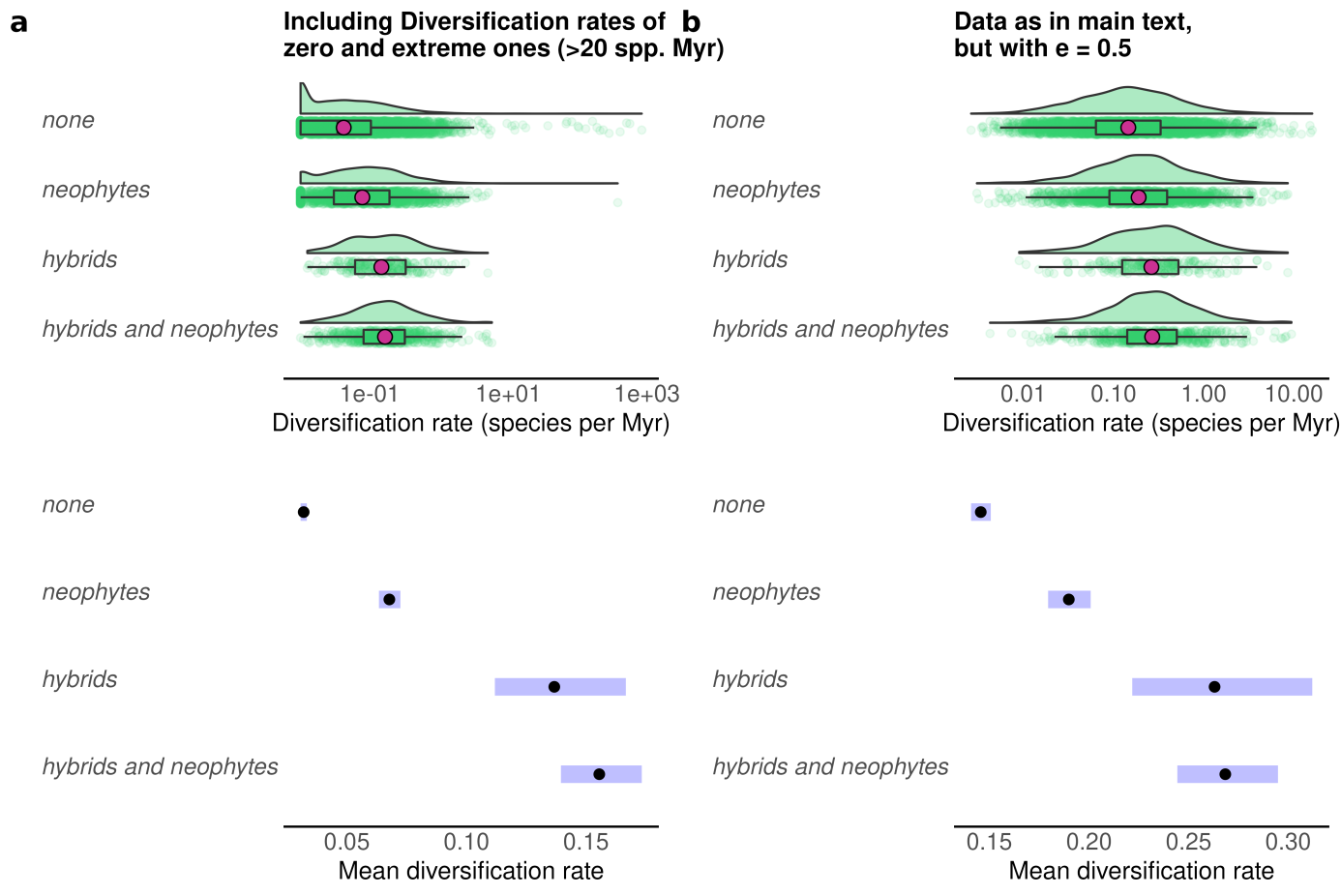


**Figure S1.** Diversification rates in relation to whether genera include hybrids and/or neophytes are robust to different methodological procedures. **(a),** Including diversification rates, D, that were extreme (> 20 spp. per Myr) or zero (genera with just one species). **(b),** Setting the parameter e (relative extinction fraction) in the formula D = log(SR_G_ * (1 - e) + e) / age, to 0.5 (in the main text e = 0.9).
